# Crystal structures of sampatrilat and sampatrilat‐Asp in complex with human ACE – a molecular basis for domain selectivity

**DOI:** 10.1111/febs.14421

**Published:** 2018-03-08

**Authors:** Gyles E. Cozier, Sylva L. Schwager, Rajni K. Sharma, Kelly Chibale, Edward D. Sturrock, K. Ravi Acharya

**Affiliations:** ^1^ Department of Biology and Biochemistry University of Bath UK; ^2^ Department of Integrative Biomedical Sciences Institute of Infectious Disease and Molecular Medicine University of Cape Town South Africa; ^3^ South African Medical Research Council Drug Discovery and Development Research Unit Department of Chemistry and Institute of Infectious Disease and Molecular Medicine University of Cape Town South Africa

**Keywords:** angiotensin‐1‐converting enzyme, crystallography, domain specificity, enzyme kinetics, enzyme structure, metalloprotease, sampatrilat

## Abstract

Angiotensin‐1‐converting enzyme (ACE) is a zinc metallopeptidase that consists of two homologous catalytic domains (known as nACE and cACE) with different substrate specificities. Based on kinetic studies it was previously reported that sampatrilat, a tight‐binding inhibitor of ACE,* K*_i_ = 13.8 nm and 171.9 nm for cACE and nACE respectively [Sharma *et al*., Journal of Chemical Information and Modeling (2016), 56, 2486–2494], was 12.4‐fold more selective for cACE. In addition, samAsp, in which an aspartate group replaces the sampatrilat lysine, was found to be a nonspecific and lower micromolar affinity inhibitor. Here, we report a detailed three‐dimensional structural analysis of sampatrilat and samAsp binding to ACE using high‐resolution crystal structures elucidated by X‐ray crystallography, which provides a molecular basis for differences in inhibitor affinity and selectivity for nACE and cACE. The structures show that the specificity of sampatrilat can be explained by increased hydrophobic interactions and a H‐bond from Glu403 of cACE with the lysine side chain of sampatrilat that are not observed in nACE. In addition, the structures clearly show a significantly greater number of hydrophilic and hydrophobic interactions with sampatrilat compared to samAsp in both cACE and nACE consistent with the difference in affinities. Our findings provide new experimental insights into ligand binding at the active site pockets that are important for the design of highly specific domain selective inhibitors of ACE.

**Database:**

The atomic coordinates and structure factors for N‐ and C‐domains of ACE bound to sampatrilat and sampatrilat‐Asp complexes (6F9V, 6F9R, 6F9T and 6F9U respectively) have been deposited in the Protein Data Bank, Research Collaboratory for Structural Bioinformatics, Rutgers University, New Brunswick, NJ (http://www.rcsb.org/).

**Abbreviations**
ACE: angiotensin‐1‐converting enzyme
AE: adverse effect
BK: bradykinin
RAAS: renin–angiotensin–aldosterone system

## Introduction

Human angiotensin‐1‐converting enzyme (ACE, http://www.chem.qmul.ac.uk/iubmb/enzyme/EC3/4/15/1.html) is a zinc dipeptidylcarboxypeptidase and an important enzyme in cardiovascular physiology 1. It plays a key role in the renin–angiotensin–aldosterone system (RAAS) by converting angiotensin I to the vasoactive peptide hormone angiotensin II 2 and also cleaves the vasodilatory peptide bradykinin (BK) further enhancing the blood pressure response 3. ACE consists of two catalytically active N‐ and C‐domains (known as nACE and cACE respectively) with similar amino acid sequence and structural topology but display different substrate specificity and inhibitor binding 4, 5, 6, 7, 8.

The ACE inhibitors such as captopril, lisinopril, enalapril, ramipril and perindopril have been widely used clinically in the treatment of hypertension and myocardial infarction as well as diabetic nephropathy. Despite the success of these ACE inhibitors, many patients (~ 25%) are unable to tolerate long‐term treatment with current‐generation ACE inhibitors because of side effects, most commonly a persistent dry cough and in some cases angioedema, a more serious adverse effect (AE) 9, 10, 11. Current approved ACE inhibitors bind both nACE and cACE with similar affinity. However, AEs due to increased plasma BK remain a major reason for drug discontinuation in certain patients. Selective inhibition of cACE has therefore been proposed to reduce circulating Ang II while still allowing nACE to attenuate the adverse accumulation of plasma BK levels 12. In contrast, selective inhibition of nACE elevates Ac‐SDKP levels in animal models 13, 14, with salutary antifibrotic and anti‐inflammatory effects. Thus, selective inhibitors of nACE could constitute a novel therapeutic class for the treatment of tissue injury and fibrosis without affecting blood pressure and with reduced AE profiles.

Sampatrilat (UK 81252, (S,S,S)‐N‐{1‐[2‐carboxy‐3‐(N‐mesyllysylamino)propyl]‐1‐cyclopentylcarbonyl}tyrosine) is a vasopeptidase or dual inhibitor of ACE and neutral endopeptidase with potential application in the treatment of hypertension and congestive heart failure. Based on its dual action, sampatrilat potentially could offer a greater benefit than traditional ACE inhibitors in the treatment of chronic heart failure 15, 16, 17. It is a hydrophilic inhibitor that contains one weakly acidic phenolic (tyrosine) group), two more strongly acidic carboxylate (tyrosine carboxylate and the central carboxylate) groups, and one strongly basic primary amine (lysine) group. In a detailed kinetic study, Sharma *et al*. 18 evaluated the *in vitro* ACE domain selectivity of sampatrilat. They observed that the inhibition of cACE (*K*_i_ = 13.8 nm) by sampatrilat was 12.4‐fold more potent than that for the nACE (*K*_i_ = 171.9 nm), indicating differences in affinities for the respective ACE domain‐binding sites (Table 1). Sharma *et al*. also reported that replacement of the P_2_ group of sampatrilat with an aspartate abrogated its cACE selectivity and lowered the potency of the inhibitor to activities in the micromolar range. The molecular basis for this selective profile was further evaluated using molecular modelling methods. It was found that the cACE selectivity of sampatrilat is due to occupation of the lysine side chain in the S_1_ and S_2_ subsites.

**Table 1:** ACE inhibitory binding constants of sampatrilat and the analogue samAsp 18

Compound	Structure	nACE *K*_i_	cACE *K*_i_	Selectivity
Sampatrilat	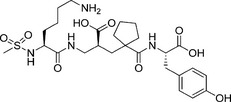	171.9 ± 32.2 nm	13.8 ± 0.2 nm	12.4
SamAsp	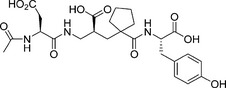	10.6 ± 0.3 μm	7.4 ± 0.3 μm	1.4

In this report we correlate the kinetic data and provide a structural basis for the sampatrilat domain selectivity using experimentally determined high‐resolution crystal structures in complex with nACE and cACE.

## Results and Discussion

### Overall structure

**Figure 1:**
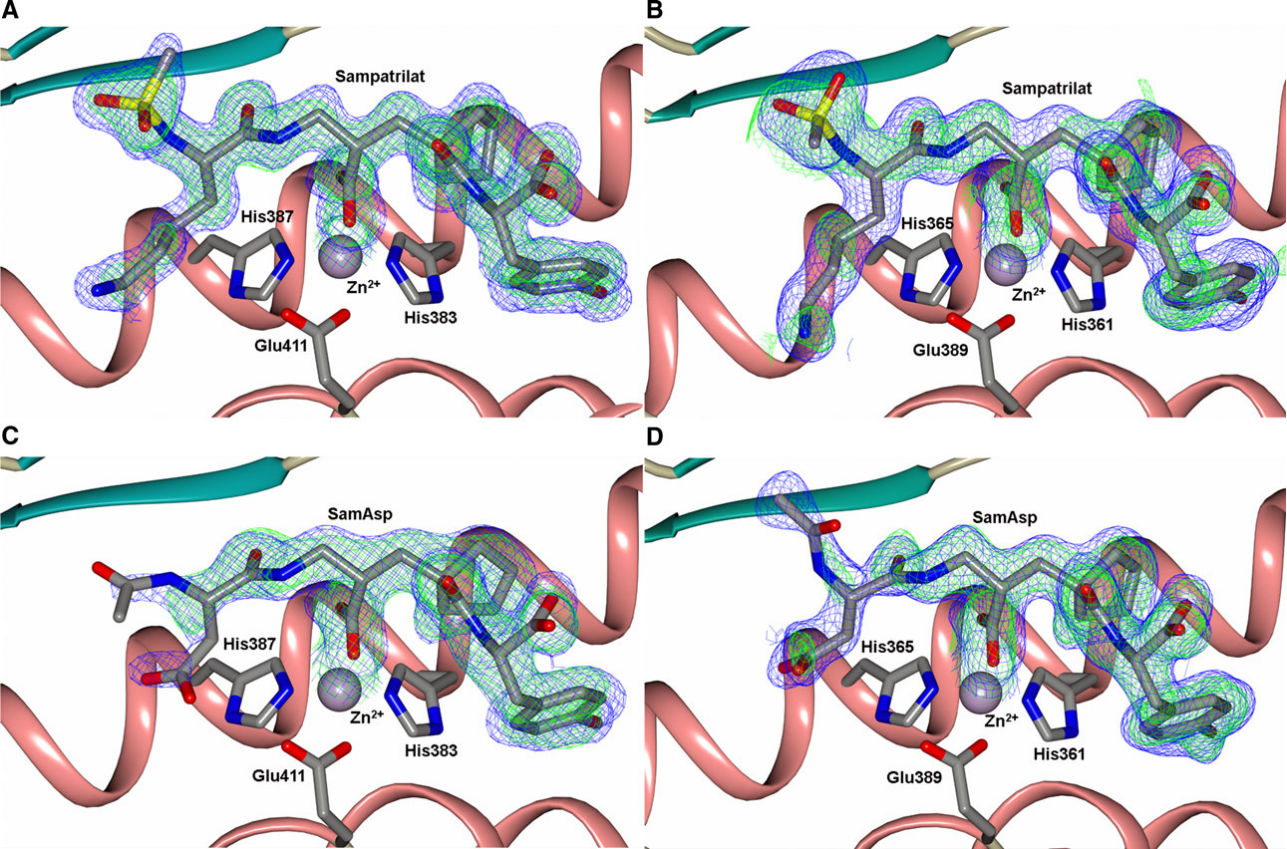
Schematic representation of inhibitors bound to the ACE domains overlayed with the final 2mFo‐DFc (blue, contoured at 1σ level) electron density map and the mFo‐DFc (green, contoured at 3σ level) electron density omit map for (A) Sampatrilat‐cACE, (B) Sampatrilat‐nACE, (C) SamAsp‐cACE and (D) SamAsp‐nACE complexes. The zinc ion is shown as a lilac sphere with the coordinating side chains shown as sticks. Alpha‐helices and β‐strands are shown in rose and dark cyan respectively.

**Figure 2:**
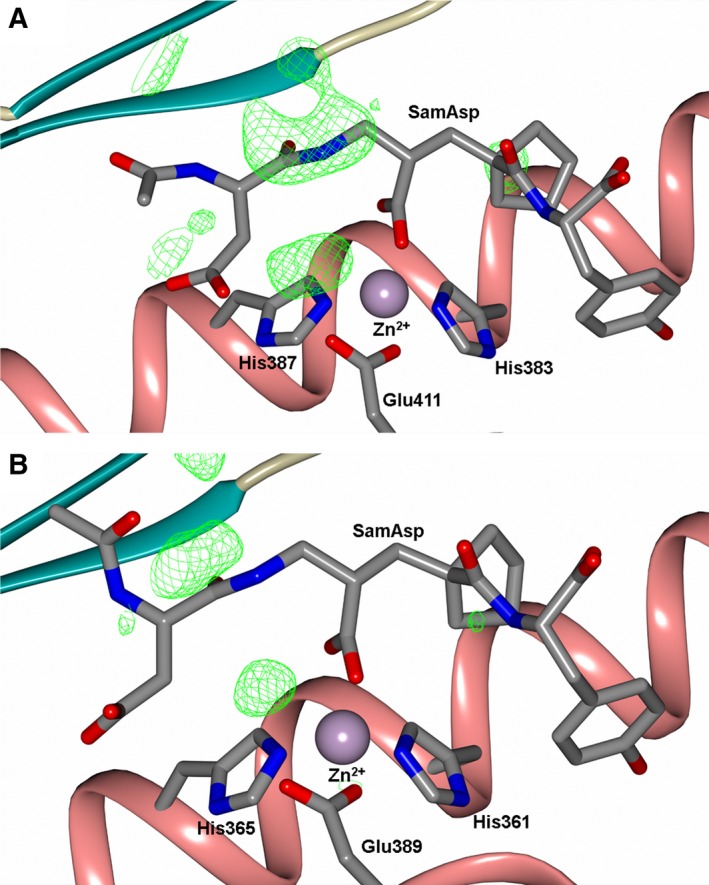
Schematic representation of (A) SamAsp‐cACE‐ and (B) SamAsp‐nACE‐binding sites overlayed with the final mFo‐DFc (green, contoured at 3σ level) electron density difference map highlighting the electron density that is maybe from an alternate conformation of the samAsp P_1_/P_2_ groups. The zinc ion is shown as a lilac sphere with the coordinating side chains shown as sticks. Alpha‐helices and β‐strands are shown in rose and dark cyan respectively.

The binding of sampatrilat and samAsp in the active sites of nACE and cACE were investigated using high resolution crystal structures (Table 2) with their respective complexes. Both the cACE structures crystallized in the usual P2_1_2_1_2_1_ space group with one protein chain in the asymmetric unit, and likewise the nACE structures crystallized in the P1 space group with two protein chains in the asymmetric unit. The overall structures for all the complexes adopt the typical ellipsoid previously observed for both nACE and cACE with the active site buried in the centre. In all four structures, the inhibitors bind in similar positions and orientations in the active site and occupy the ACE inhibitor recognition subsites S_2_, S_1_, S_1_ʹ and S_2_ʹ (Fig. 1). There is an extensive set of interactions throughout these subsites and with the catalytic Zn^2+^ ion deep inside the central cavity of the protein molecule. While the electron density for sampatrilat in both cACE and nACE is well defined and unambiguous, samAsp in both domains shows some flexibility in the S_1_ and S_2_ subsites. The electron density for this part is not as well defined as for the rest of the molecule, and in both domains there is some additional density located next to the P_1_ backbone nitrogen that indicates a potential second conformation, although the density is not sufficient to model this (Fig. 2). The interaction details for sampatrilat and the predominant conformation of samAsp are described below.

**Table 2:** Crystallographic data collection and structure refinement statistics. Inner shell, overall and outer shell statistics are given in square brackets, unbracketed and round brackets respectively

	tACE Sampatrilat	tACE SampatrilatASP	nACE Sampatrilat	nACE SampatrilatASP
Resolution (Å)	[84.76–8.75] (1.63–1.60)	[85.86–9.11] (1.94–1.90)	[74.35–9.26] (1.72–1.69)	[56.01–10.12] (1.88–1.85)
Space group	P2_1_2_1_2_1_	P2_1_2_1_2_1_	P1	P1
Cell dimensions (a,b,c) angles (α,β,γ)	57.2, 84.8, 134.1 Å 90.0, 90.0, 90.0°	56.3, 85.9, 133.2 Å 90.0, 90.0, 90.0°	73.2, 77.2, 83.1 Å 88.4, 64.2, 75.3°	72.6, 77.0, 82.7 Å 88.4, 64.3, 75.4°
Molecules/asymmetric unit	1	1	2	2
Total/Unique reflections	428 984 85 788	1 295 115 51 795	1 177 792 171 761	566 485 131 017
Completeness (%)	[99.9] 98.8 (87.5)	[99.9] 100.0 (100.0)	[99.4] 97.2 (95.8)	[99.3] 98.4 (92.9)
*R*_merge_	[0.047] 0.096 (0.572)	[0.057] 0.382 (6.783)	[0.030] 0.045 (0.395)	[0.054] 0.092 (1.202)
*R*_pim_	[0.033] 0.069 (0.478)	[0.016] 0.110 (2.121)	[0.019] 0.028 (0.260)	[0.045] 0.075 (0.959)
<I/σ(I)>	[23.5] 8.2 (1.5)	[26.6] 9.3 (1.3)	[40.3] 17.9 (3.6)	[20.2] 7.7 (1.5)
CC_1/2_	[0.996] 0.996 (0.628)	[0.999] 0.997 (0.576)	[0.998] 0.998 (0.950)	[0.995] 0.994 (0.512)
Multiplicity	[4.7] 5.0 (2.9)	[22.0] 25.0 (21.7)	[6.3] 6.9 (6.4)	[4.4] 4.3 (4.4)
Refinement statistics				
*R*_work/_ *R*_free_	0.165/0.187	0.167/0.209	0.183/0.212	0.198/0.228
Rmsd in bond lengths (Å)	0.009	0.007	0.010	0.006
Rmsd in bond angles (°)	1.058	0.848	1.069	0.802
Ramachandran statistics (%)				
Favoured	98.5	98.1	98.2	97.8
Allowed	1.3	1.9	1.6	2.0
Outliers	0.2	0.0	0.2	0.2
Average B‐ factors (Å^2^)				
Protein	22.6	34.7	38.9	39.8
Ligand	38.5	48.1	49.1	50.4
Water	30.5	36.1	40.5	37.6
Number of atoms				
Protein	9578	9448	20 940	20 222
Ligand	452	352	699	758
Water	686	344	962	748
PDB code	6F9T	6F9U	6F9V	6F9R

**Figure 3:**
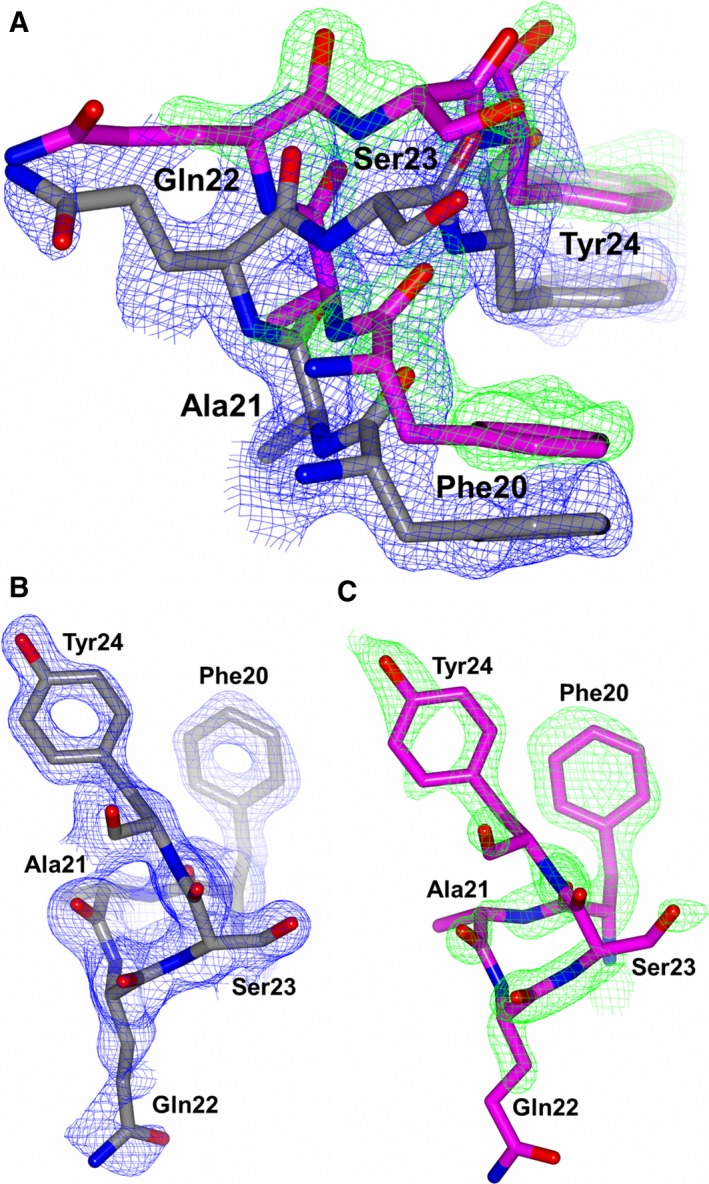
Residues 20–24 of nACE from the sampatrilat complex structure to highlight the second conformation of part of the nACE hinge region observed. (A) Both conformations, (B) Predominant conformation and (C) Alternate conformation overlayed with the 2mFo‐DFc (blue, contoured at 1σ level) and mFo‐DFc (green, contoured at 3σ level) electron density omit maps.

**Figure 4:**
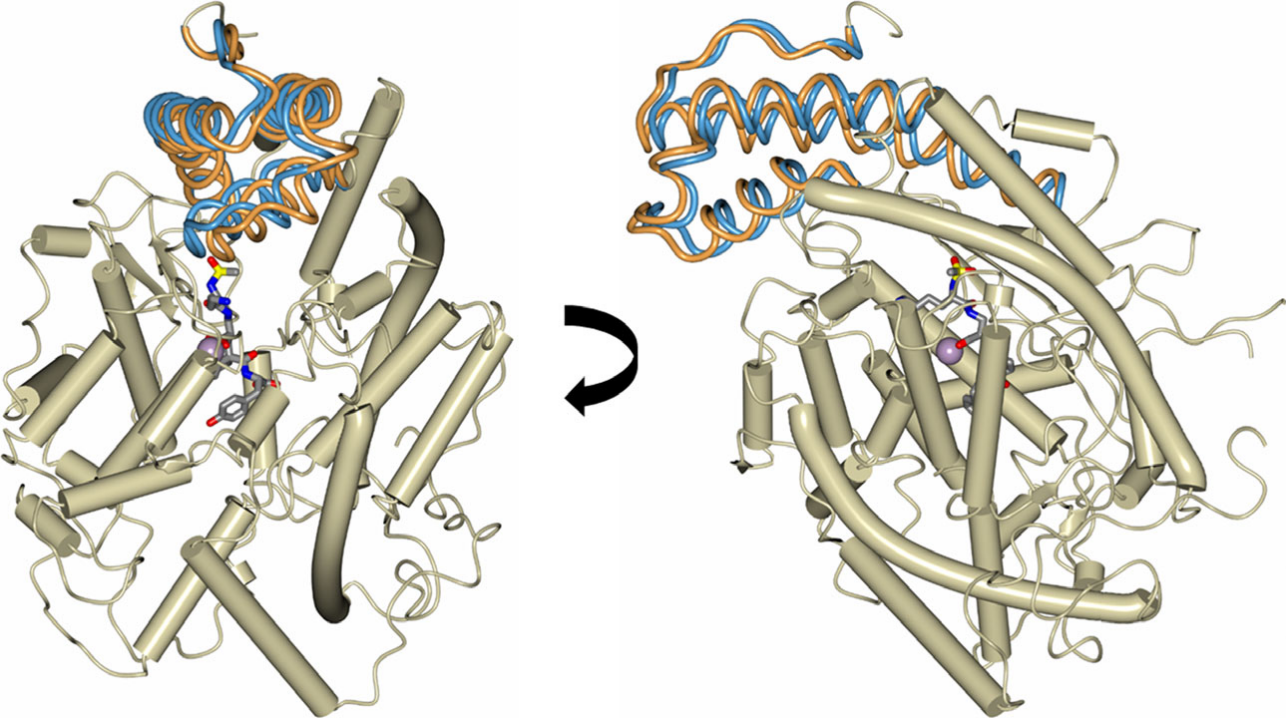
Two views of a schematic representation of the nACE protein chain from the sampatrilat complex structure showing the two conformations of part of the hinge region (residues 4–43 and 57–99) in fat worm style (predominant and alternate conformations shown in orange and blue respectively). Alpha‐helices are shown as tubes, sampatrilat as sticks and the zinc ion as a lilac sphere.

Residues 1–100 contains the N‐terminal hinge region of ACE which consists of the α1, α2 and α3 helices that forms a lid‐like structure that may restrict access of large polypeptides into the active site 19. In the nACE structures, sections of this region often show flexibility with increased temperature factors (B‐factors) and poor electron density. This is typically more apparent in one chain of the asymmetric unit, and this region has a small, up to 3Ẳ, shift between the two chains 20. Both the sampatrilat and samAsp nACE structures exhibit this flexibility in the hinge region. In one chain of the asymmetric unit, residues 4–43 and 57–99 of sampatrilat/nACE, and 10–28 and 75–99 of samAsp/nACE showed some additional density in the mFo‐DFc electron density map indicating a second conformation for these regions (Fig. 3). While this extra density was not complete for every residue in these ranges, overall it was sufficient to model a second conformation for this part of the molecule (Fig. 4). This second conformation is equivalent to the conformation observed in the other protein chain. While this is not a new conformation for the hinge region, this represents the first time that the flexibility of this region has been so clearly observed within one chain of the asymmetric unit.

Both ACE activity, and inhibitor affinity for ACE have previously been shown to be dependent on chloride ions, with cACE exhibiting this to a greater extent than nACE 8, 21, 22, 23, 24. As observed with previous structures, all the complexes described here contain the usual two chloride ions bound to cACE, and one to nACE. These are the active forms of the enzymes. The first chloride‐binding site in cACE contains interactions with R186, W485, R489 and a water molecule, nACE does not bind an equivalent chloride ion. The second chloride binding site in cACE contains interactions with Y224, R522, and a water molecule. This site is conserved in nACE, with equivalent residues Y202 and R500.

### cACE cysteine‐496 residue

**Figure 5:**
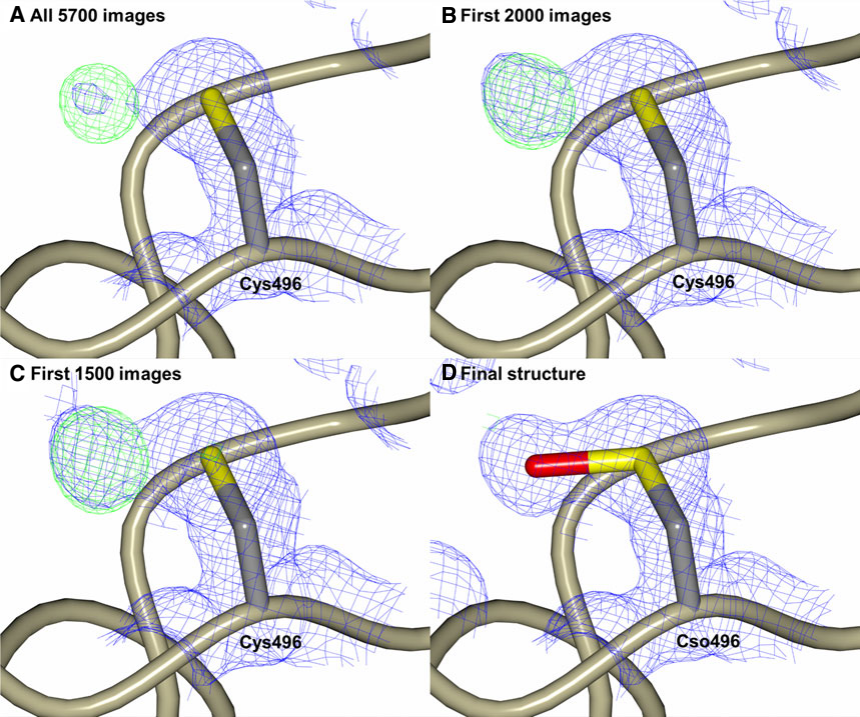
Modification of Cys496 from the tACE‐sampatrilat complex structure. (A,B) and (C) show the 2mFo‐DFc (blue, contoured at 1σ level) and mFo‐DFc (green, contoured at 3σ level) electron density omit maps generated from the first 5700, 2000 and 1500 images respectively. (D) The modelled oxidized cysteine residue Cso (S‐Hydroxycysteine) overlayed with the final 2mFo‐DFc (blue, contoured at 1σ level) electron density map. There is no density visible around this residue in the mFo‐DFc difference electron density map contoured at 3σ level indicating the oxidized cysteine fits the electron density map.

Closer examination of the electron density map for the sampatrilat‐cACE complex structure showed a patch of electron density adjacent to Cys496 that was too close to be interpreted as a water molecule (Fig. 5A), therefore, suggesting a modification of the cysteine residue. This density is not visible in the samAsp‐cACE complex structure, or the nACE complex structures. However, reviewing previously deposited cACE structures suggests that there is often a smaller area of equivalent density in the mFo‐DFc difference electron density map. In order to determine if this density was caused due to the effect of radiation on the cysteine residue during data collection, the X‐ray data processing was performed using the first 2000 and then the first 1500 images (Fig. 5B,C). This showed that the density was more apparent with reduced exposure to X‐ray radiation, thereby indicating that the radiation caused the removal of the cysteine modification, rather than forming it. This may be partly why the modification has not been observed, or considered in previously reported crystal structures. The cysteine modification in the present structure is most likely to be an oxidation resulting in S‐hydroxycysteine, which could be modelled well into the electron density (Fig. 5D). It is unclear what causes this modification and if it has any role in ACE function, or whether it is simply an artefact of the crystallization process. A previously published report suggested that Cys496 in tACE was present in the reduced form as indicated by labelling with 5‐[[2‐(iodoacetyl)amino]ethylamino]naphthalene‐1‐sulphonic acid 25. This cysteine is conserved in nACE (Cys474), and is the only cysteine residue in each domain that is not involved in intramolecular disulphide bond formation. It is interesting to note that this cysteine has been suggested to be involved in dimerization of ACE through the C‐domain via intermolecular disulphide bond formation 26.

### Inhibitor‐binding sites

**Figure 6:**
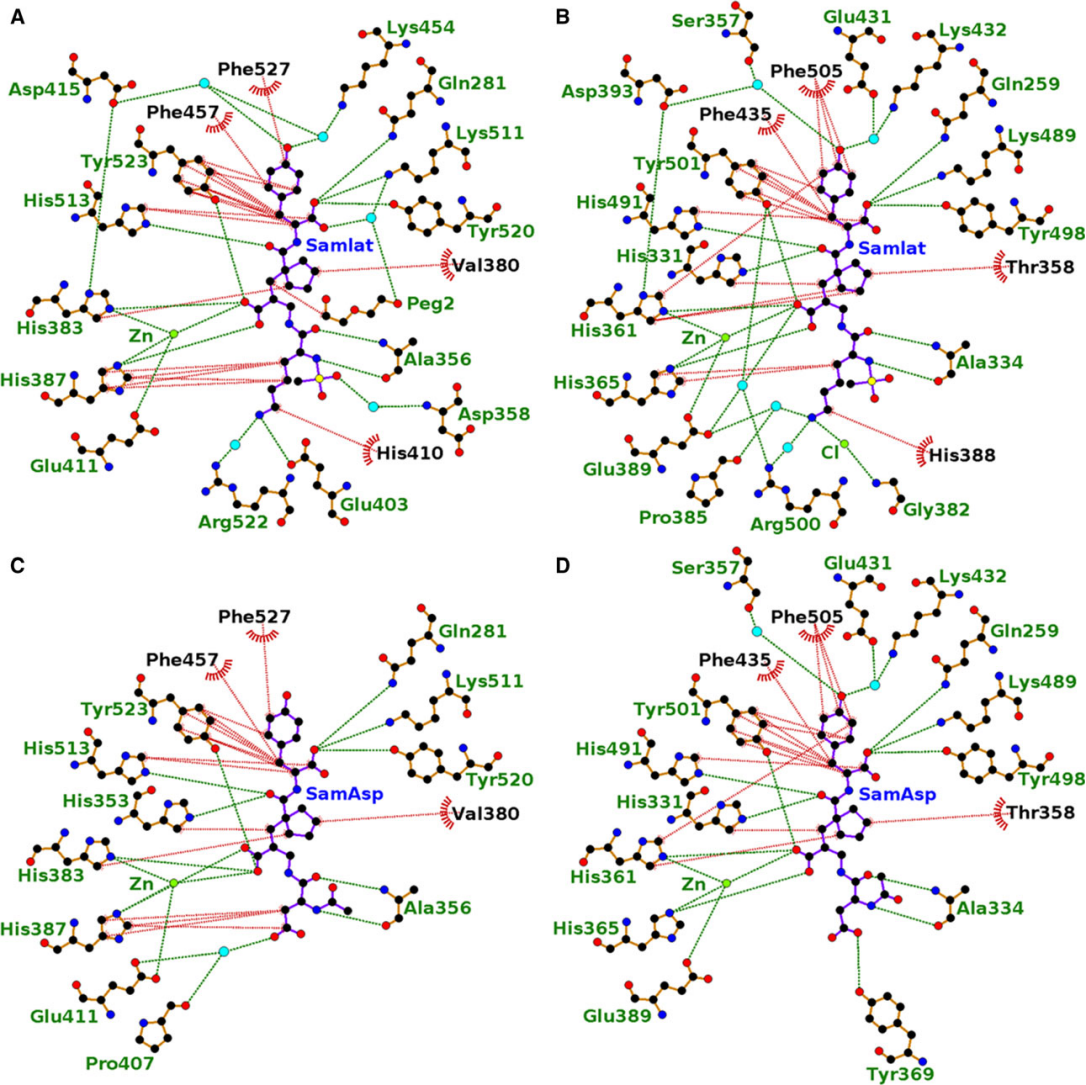
Ligplot representation of the binding site interactions of (A) sampatrilat‐cACE, (B) sampatrilat‐nACE, (C) samAsp‐cACE and (D) samAsp‐nACE. H‐bond/electrostatic interactions are shown in green, hydrophobic interactions in red and water molecules as cyan spheres. Residues solely involved in hydrophobic interactions are represented by red, semicircular symbols.

It is well established that both nACE and cACE contain similar active site structures 27 yet they possess distinct substrate specificities and chloride activation profile 24. In particular, the selectivity of different ACE inhibitors could be correlated with the differences in amino acid residues between these individual domains at the active site. A detailed distribution of residues involved in sampatrilat/samAsp recognition in nACE and cACE complexes are listed in Table 3 (Fig. 6).

**Table 3:** Comparison of amino acid residues involved in cACE and nACE interactions with sampatrilat and samAsp and with previously established interactions of cACE‐specific phosphinic inhibitor RXPA380 30

Sampatrilat		SamAsp		RXPA380
C‐domain sACE	N‐domain sACE	C‐domain sACE	N‐domain sACE	C‐domain sACE
Direct hydrogen bond interactions				
Gln281	Gln259	Gln281	Gln259	Gln281
–	His331	His353	His331	His353
Ala356	Ala334	Ala356	Ala334	Ala356
His383	His361	His383	His361	His383
His387	His365	His387	His365	His387
–	–	–	Tyr369	–
Glu403	–	–	–	–
Lys511	Lys489	Lys511	Lys489	Lys511
His513	His491	His513	His491	His513
Tyr520	Tyr498	Tyr520	Tyr498	Tyr520
Tyr523	Tyr501	Tyr523	Tyr501	Tyr523
Water‐/ion‐mediated interactions				
–	–	–	–	Gln281
PEG2	–	–	–	–
Asp368	–	–	–	–
–	Ser357	–	Ser357	–
–	Gly382	–	–	–
–	Pro385	Pro407	–	–
–	Glu389	Glu411	–	–
Asp415	Asp393	–	–	–
–	Glu431	–	Glu431	–
Lys454	Lys432	–	Lys432	–
Lys511	–	–	–	Lys511
Arg522	Arg500	–	–	–
Hydrophobic interactions				
PEG2	His331	His353	His331	His353
–	–	–	–	Ala354
–	–	–	–	Ser355
Val380	Thr358	Val380	Thr358	Val380
His383	His361	His383	His361	His383
His387	His365	His387	–	His387
–	–	–	–	Phe391
His410	His388	–	–	His410
Phe457	Phe435	Phe457	Phe435	Phe457
–	–	–	–	Phe512
His513	His491	His513	His491	His513
–	–	–	–	Val518
Tyr523	Tyr501	Tyr523	Tyr501	Tyr523
Phe527	Phe505	Phe527	Phe505	Phe527

With the zinc binding, P_1_ʹ and P_2_ʹ groups being identical in both sampatrilat and samAsp, it is not surprising that the majority of interactions in this region are conserved in all four structures, and are typical based on other ligand and inhibitor complexes with cACE and nACE. The catalytic zinc ion is at the centre of a highly coordinated system involving His383, His387 and Glu411 of cACE (His361, His365 and Glu389 in nACE) and a potential bidendate interaction with the zinc‐binding carboxylate group of the inhibitors, although one oxygen of the carboxylate group is always more ideally positioned. There is also a conserved H‐bond between the zinc binding group and a tyrosine residue (Tyr523 in cACE; Tyr501 in nACE). The P_1_ʹ backbone carbonyl binds to His513 and His491 in cACE and nACE respectively. Gln281, Lys511 and Tyr520 of cACE (Gln259, Lys489 and Tyr498 of nACE) all interact with the P_2_ʹ terminal carboxylate. In addition, many hydrophobic interactions with the P_1_ʹ and P_2_ʹ groups from cACE residues Val380, His383, His387, Phe457, His513, Tyr523 and Phe527 (Thr358, His361, His365, Phe435, His491, Tyr501 and Phe505 in nACE) are also conserved.

The differences between the structures in the S_1_ʹ and S_2_ʹ subsites are largely involving water‐mediated interactions. In both nACE complex structures, and the sampatrilat‐cACE complex structure, there are two water molecules adjacent to the P_2_ʹ tyrosine group. However, there are variations in the protein residues involved in these water‐mediated interactions (Fig. 3 and Table 2 for details) caused by small shifts in inhibitor and water positions. The samAsp‐cACE complex has no water molecules visible near the inhibitor tyrosine group. The other difference between the structures is the orientation of the His353 side chain in the sampatrilat‐cACE structure. In the other three structures, this residue (His331 in nACE) forms a H‐bond and hydrophobic interaction with the P_1_ʹ backbone and backbone carbonyl, respectively, of the inhibitors. In the sampatrilat‐cACE structure, His353 is orientated differently and in the vacated space, a poly(ethylene glycol) molecule is bound. This mimics the hydrophobic interaction seen in the other structures and forms a water‐mediated interaction with the sampatrilat C‐terminal carboxylate.

**Figure 7:**
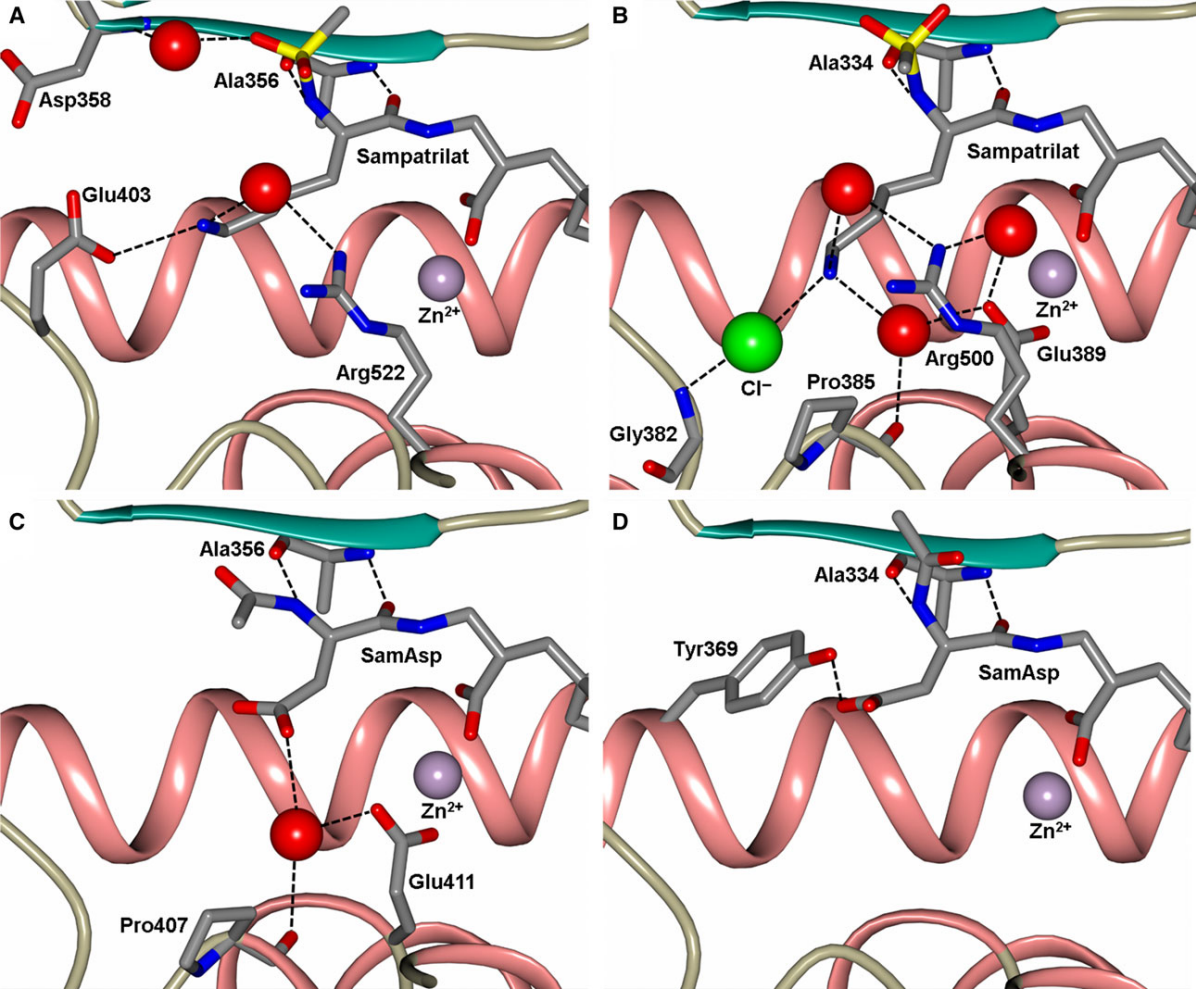
Close up views of (A) sampatrilat‐cACE, (B) sampatrilat‐nACE, (C) samAsp‐cACE and (D) samAsp‐nACE binding sites showing the H‐bond/electrostatic interactions in the S_1_ and S_2_ subsites. The protein chain is shown as a cartoon with α‐helices and β‐strands in rose and dark cyan, respectively, and water molecules are depicted as red spheres.

With the P_1_/P_2_ groups being different between sampatrilat and samAsp, it is unsurprising that the largest differences between the structures are observed in the S_1_ and S_2_ subsites (Fig. 7). The only identical interactions between all four structures are from the backbone nitrogen and carbonyl of cACE Ala356 (Ala334 in nACE) and the conserved backbone nitrogen and carbonyl mimic of sampatrilat/samAsp. In addition, in both sampatrilat structures, the methanesulphonamide group is surrounded by water molecules. These water molecules are not ideally positioned to form water‐mediated interactions with specific residues, and it is difficult to predict the rotation of the methanesulphonamide group based on the electron density, but it is likely that the solvated environment around this group helps to stabilize the bound sampatrilat molecule. The rotational orientations of the methanesulphonamide group in the cACE and nACE sampatrilat structures were chosen based on lowest overall B‐factors for the atoms, but it is likely that this group has some degree of rotational freedom in both structures.

The cACE‐sampatrilat structure has extensive hydrophobic interactions from His387 and His410 with the lysine sidechain of the inhibitor, and this lysine side chain also has a water‐mediated interaction with Arg522 and a direct H‐bond with Glu403 (Figs 6A and 7A). Therefore, the lysine side chain is strongly bound in the S_1_ and S_2_ subsites. In comparison, the cACE‐samAsp structure retains some hydrophobic interactions with His387, and has a single water‐mediated interaction with Pro407 and Glu411 (Figs 6C and 7C).

In nACE, sampatrilat has hydrophobic interactions with His365 and His388 equivalent to those seen in cACE‐sampatrilat complex, but they are less extensive due to a small shift in the orientation of the lysine side chain of sampatrilat (Figs 6B and 7B). It retains the water‐mediated interaction with Arg500, and has an additional water‐mediated interaction with Pro385 and Glu389. Also adjacent to the lysine side chain nitrogen is a region of electron density that is too strong for a water molecule and has been modelled as a chloride ion that is additionally bound to the backbone nitrogen of Gly382. SamAsp lacks hydrophobic interactions with the P_1_/P_2_ part of the molecule, it, however, has a direct H‐bond from the aspartate group with Tyr369 (Figs 6D and 7D).

### Comparison of binding interactions

A previous study compared the affinity and selectivity of sampatrilat and samAsp for cACE and nACE 18. This reported that sampatrilat inhibited both cACE and nACE in the low nanomolar range, but it was 12.4‐fold more selective for cACE (*K*_i_ of 13.8 and 171.9 nm respectively). In comparison, samAsp showed no selectivity, and was also less potent with micromolar *K*_i_ values for both cACE and nACE. The assays were performed in the presence of 200 mm NaCl, ensuring bound chloride ions to give fully active enzyme, and maximal affinity for the inhibitors. As noted earlier, the crystal structures contained bound chloride ions, indicating the active form.

Comparison of the binding interactions of sampatrilat and samAsp in cACE and nACE in part explains the differences in affinity. As described above, the interactions with the zinc‐binding P_1_ʹ and P_2_ʹ groups of the inhibitors are largely conserved in all the structures with the only significant difference likely to be the lack of water‐mediated interactions with the P_2_ʹ tyrosine group of samAsp in cACE. This could potentially contribute to reduced affinity for samAsp, but in general the main differences in binding interactions were observed in the S_1_ and S_2_ subsites. In both cACE and nACE there was a reduction in the number of interactions in these subsites for samAsp compared to sampatrilat. In particular, for cACE, there is a reduced number of hydrophobic interactions and significant loss of binding to Glu403, which explains why cACE binds sampatrilat with a much higher affinity than samAsp. In nACE, while Tyr369 provides a direct H‐bond with samAsp, there are much more extensive interactions with the lysine side chain of sampatrilat, which is again consistent with the different affinities of these inhibitors.

Comparison of sampatrilat binding with the ACE domains shows a lack of any direct H‐bond interaction between the lysine side chain of sampatrilat and nACE (due to Glu403 of cACE being replaced by Arg381 in nACE), and a less extensive hydrophobic network. This is likely to contribute to the lower affinity of sampatrilat for nACE compared to cACE. However, it also needs to be considered that a previous study with nACE‐specific RXP407 and 33RE inhibitors indicated that a P_2_ʹ carboxylic acid in place of an amide group greatly reduces, or even abolishes N‐domain specificity 28, 29. Further experimental analysis is required to clarify whether or not this is a general rule for all inhibitors, which could be contributing to the cACE selectivity of sampatrilat even though the interactions with the P_2_ʹ carboxylic acid are conserved in both domains.

### Comparison with sampatrilat/samAsp molecular modelling results

The study measuring the affinity of sampatrilat/samAsp for cACE and nACE also included molecular modelling studies (docking and molecular dynamics) 18. The docking studies predicted two potential orientations of sampatrilat in both domains, with the inhibitor lysine side chain located in either the S_1_ or S_2_ subsites. The zinc binding P_1_ʹ and P_2_ʹ groups showed good agreement between the docking, molecular dynamics and crystal structures for both cACE and nACE complexes, albeit the water‐mediated interactions shown in the crystal structures were predicted to be direct interactions in the molecular modelling study. The sampatrilat lysine orientated in the S_1_ subsite interacted with Glu143 in cACE and the nonequivalent Asp43 in nACE, and shows some flexibility during molecular dynamics studies, which showed additional interactions with other negatively charged residues in the S_1_ subsite. There is no evidence for this orientation of sampatrilat in the crystal structures. In contrast, the S_2_ subsite docked orientation showed good agreement with the crystal structures presented here, including the direct H‐bond with Glu403 in cACE. However, the molecular dynamics study predicted movement away from the docked poses, which is inconsistent with the crystal structures. This is probably because there are important water‐/ion‐mediated interactions in the crystal structures, which are not accounted for in the molecular modelling study 18.

The molecular modelling analysis for samAsp predicted electrostatic interactions between the inhibitor aspartate and Arg522 of cACE and the equivalent Arg500 of nACE. These interactions are not observed in the crystal structures. Additionally the H‐bond between samAsp and Tyr369 shown in the crystal structure was not observed during the molecular modelling studies. However, the crystal structures of the samAsp complexes show that the P_1_/P_2_ part of the molecule is flexible, indicating other interactions could be possible, although they are unlikely to be strong.

### Comparison with cACE‐specific RXPA380 inhibitor

**Figure 8:**
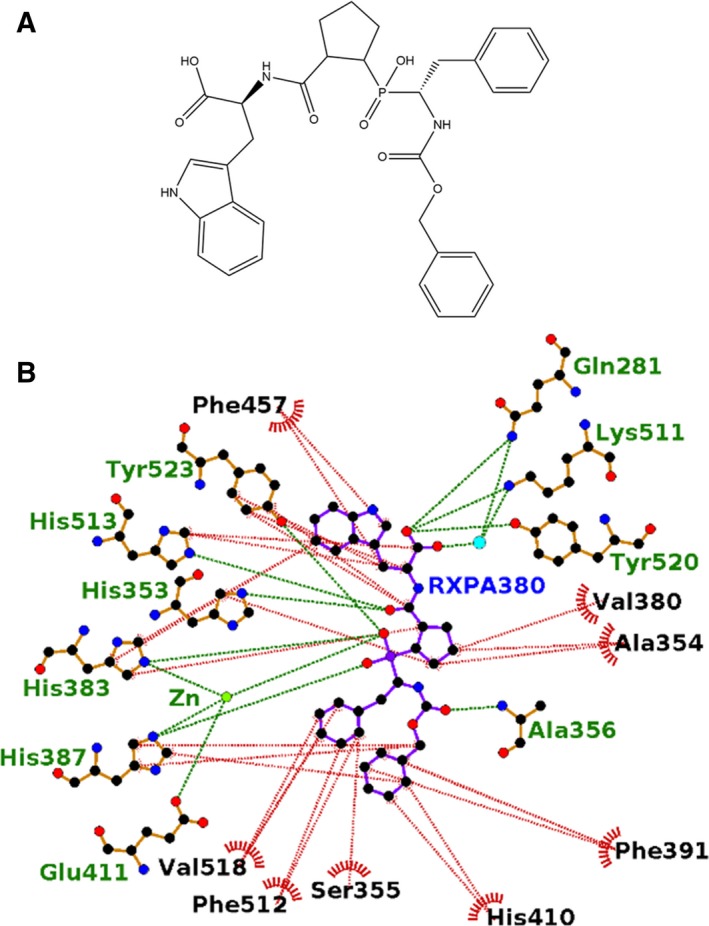
(A) Structure of RXPA380. (B) Ligplot representation of the binding site interactions of RXPA380 in cACE. H‐bond/electrostatic interactions are shown in green, hydrophobic interactions in red and water molecules as cyan spheres.

RXPA380 (Fig. 8A) is a cACE‐specific inhibitor exhibiting a 3000‐fold lower *K*_i_ than for nACE 30. This has been attributed to the increased hydrophobicity of the cACE S_1_ʹ and S_2_ʹ subsites to accommodate the P_1_ʹ and P_2_ʹ proline and tryptophan of RXPA380, and hydrophobic interactions between the RXPA380 P_2_ phenylalanine and cACE Phe391 (Fig. 8B). In nACE, the equivalent residue is Tyr369, which is not only more hydrophilic but also an equivalent binding orientation of RXPA380 in nACE is not possible due to the hydroxyl group of Tyr369 only being 2 Ẳ away from the inhibitor phenyl ring.

A comparison of the sampatrilat and RXPA380 inhibitor structures and their interactions in the ACE complex structures (Table 3) show that nearly all of the direct H‐bond interactions are conserved even though the inhibitor structures differ. The notable difference is the cACE interaction between Glu403 and sampatrilat P_2_ lysine. As described above, the RXPA380 selectivity for cACE was attributed to a large number of hydrophobic interactions, whereas with sampatrilat the difference between binding to cACE and nACE is mainly due to a direct binding to Glu403 which is replaced by an arginine in the N domain.

## Conclusions

The high‐resolution X‐ray crystal structures presented here demonstrate the detailed interactions involved in the binding of sampatrilat and samAsp to both cACE and nACE. In addition the flexibility of the nACE hinge region could be modelled in the form of two conformations within one chain of the asymmetric unit for both sampatrilat and samAsp nACE complex structures, and an oxidized form of cACE Cys496 was observed for the first time. Further work is needed to determine the significance of this cysteine modification, or whether it is an artefact of the crystallization process. This cysteine residue has been implicated in the dimerization of ACE through the C‐domain by formation of an intermolecular disulphide bond. The S‐hydroxycysteine indicates the reactivity of this residue, which is consistent with the dimerization theory.

It has been shown that sampatrilat is a higher affinity inhibitor for both cACE and nACE than the samAsp analogue. The results from the crystal structures clearly show that there are many more interactions with sampatrilat than samAsp in the S_1_ and S_2_ subsites of both domains, thereby explaining the difference in affinities. Sampatrilat shows a 12.4‐fold specificity for cACE over nACE. In the crystal structures, the interactions involving the zinc binding, P_1_ʹ and P_2_ʹ groups of sampatrilat are almost identical in cACE and nACE. In the nACE S_1_ and S_2_ subsites, there are hydrophobic and water‐/ion‐mediated interactions with the lysine group of sampatrilat. By comparison, in cACE there are a greater number of hydrophobic interactions and a direct H‐bond with Glu403 (Arg381 in nACE), which at least partly explains the difference in affinity of sampatrilat for the two domains. However, the previous observation that nACE has a higher affinity for P_2_ʹ terminal amides over carboxylates also has to be considered, and this could contribute to the lower affinity for sampatrilat, which contains a terminal carboxylate.

A comparison of sampatrilat and the highly (3000‐fold) specific inhibitor RXPA380 binding to cACE shows a difference in the reason for the specificity. While sampatrilat's specificity is caused by a mixture of hydrophobic interactions and a direct H‐bond with Glu403 of cACE, in contrast, RXPA380 gains its specificity exclusively from a large number of hydrophobic interactions with its tryptophan side chain in the S_2_ʹ subsite, and the phenyl groups in the S_1_ and S_2_ subsites. This difference in binding attributes suggests the potential to create an inhibitor with greater affinity and specificity that retains a lysine‐like moiety that can target the nonconserved Glu403 but includes both a P_2_ʹ tryptophan like group and phenyl groups targeting the hydrophobic residues in the S_2_ʹ, S_1_ and S_2_ subsites. A full structure–activity relationship (SAR) study targeting these different interactions would identify the relative importance of them for inhibitor affinity to, and specificity for cACE.

## Experimental methods

### Enzymes and inhibitors

Minimally glycosylated N‐ and C‐domain human ACE proteins (N389 and G13 respectively) were generated by expression in cultured mammalian CHO cells and purified to homogeneity as described previously 20, 31. Synthesis and inhibition data for sampatrilat and samAsp analogue have been reported previously 18.

### X‐ray crystallographic studies

The ACE domains were preincubated with either sampatrilat or samAsp in a 4:1 v/v ratio of protein (8 mg·mL^−1^ cACE and 5 mg·mL^−1^ nACE both in 50 mm Hepes, pH 7.5, 0.1 mm PMSF) and 20 mm inhibitor for 1 hour (cACE on ice, nACE at room temperature). Cocrystals were obtained with 1 μL of the protein‐inhibitor complex mixed with equal volume of reservoir solution (0.1 m MIB buffer pH 4.0, 5% glycerol and 15% poly(ethylene glycol) 3350 for cACE with both sampatrilat and samAsp, and 30% poly(ethylene glycol) 550 MME/poly(ethylene glycol) 20000, 0.1 m Tris/Bicine pH 8.5, and 60 mm divalent cations, Molecular Dimensions Morpheus A9 for nACE with both sampatrilat and samAsp) and suspended above the well as a hanging drop.

X‐ray diffraction data were collected on station IO3 (cACE/sampatrilat, cACE/samAsp and nACE/sampatrilat) and I24 (nACE/samAsp) at the Diamond Light Source (Didcot, UK). Crystals were kept at constant temperature (100 K) under the liquid nitrogen jet during data collection. Images were collected using a PILATUS3‐6M detector (Dectris, Switzerland). Raw data images were indexed and integrated with DIALS 32, and then scaled using AIMLESS 33 from the CCP4 suite 34. Initial phases were obtained by molecular replacement with PHASER 35 using PDB code 1O8A 19 for cACE and 3NXQ 20 for nACE as the search models. Further refinement was initially carried out using REFMAC5 36 and then Phenix 37, with COOT 38 used for rounds of manual model building. Ligand and water molecules were added based on electron density in the Fo‐Fc Fourier difference map. MolProbity 39 was used to help validate the structures. Crystallographic data statistics are summarized in Table 2. All figures showing the crystal structures were generated using CCP4 mg 40 and schematic binding interactions are displayed using Ligplot+ 41.

## Conflict of interest

The authors declare no competing financial interests.

## Author contributions

GEC performed all the crystallography experiments, analysed the data and wrote the manuscript. SLS and RKS carried out all the protein expression and kinetics studies. EDS and KC supervised the biochemical work, analysed the data and edited the manuscript. KRA supervised the structural study, analysed the data and edited the manuscript. All authors reviewed the manuscript.
